# Detecting androgen receptor (AR), AR variant 7 (AR-V7), prostate-specific membrane antigen (PSMA), and prostate-specific antigen (PSA) gene expression in CTCs and plasma exosome-derived cfRNA in patients with metastatic castration-resistant prostate cancer (mCRPC) by integrating the VTX-1 CTC isolation system with the QIAGEN AdnaTest

**DOI:** 10.1186/s12885-024-12139-3

**Published:** 2024-04-16

**Authors:** Haiyan E. Liu, Meghah Vuppalapaty, Christian R. Hoerner, Colin P. Bergstrom, Michael Chiu, Clementine Lemaire, James Che, Amanpreet Kaur, Adam Dimmick, Sean Liu, Thomas J. Metzner, Menna Araya, Steve Crouse, Markus Sprenger-Haussels, Martin Schlumpberger, John T. Leppert, Siegfried Hauch, Elodie Sollier, Alice C. Fan

**Affiliations:** 1 Vortex Biosciences, Inc, Pleasanton, CA USA; 2 Division of Oncology, Department of Medicine, Stanford University School of Medicine, Stanford, CA USA; 3 Department of Urology, Stanford University School of Medicine, Stanford, CA USA; 4 Stanford Comprehensive Cancer Center, Stanford University School of Medicine, Stanford, CA USA; 5 QIAGEN GmbH, Hilden, Germany

**Keywords:** Prostate cancer, Castration resistance, Androgen receptor inhibitors, Therapeutic resistance, AR-V7, PSMA, Circulating tumor cells, Exosomes, Cell-free RNA

## Abstract

**Background:**

Therapies for metastatic castration-resistant prostate cancer (mCRPC) include targeting the androgen receptor (AR) with androgen receptor inhibitors (ARIs) and prostate-specific membrane antigen (PSMA). Having the ability to detect AR, AR splice variant 7 (AR-V7), or PSMA in circulating tumor cells (CTCs) or circulating exosomal cell-free RNA (cfRNA) could be helpful to guide selection of the appropriate therapy for each individual patient. The Vortex Biosciences VTX-1 system is a label-free CTC isolation system that enables the detection of the expression of multiple genes in both CTCs and exosomal cfRNA from the same blood sample in patients with mCRPC. Detection of both AR-V7 and PSMA gene expression in both CTCs and cfRNA simultaneously has not yet been reported.

**Methods:**

To characterize the combined VTX-1-AdnaDetect workflow, 22Rv1 cancer cells were spiked into blood from healthy donors and processed with the VTX-1 to mimic patient samples and assess performances (capture efficiency, purity, AR and AR-V7 expression). Then, we collected 19 blood samples from 16 patients with mCRPC and therapeutic resistance to androgen receptor inhibitors (ARIs). Plasma was separated and the plasma-depleted blood was processed further with the VTX-1 to collect CTCs. Both plasma exosomal cfRNA and CTCs were subsequently analyzed for AR, AR-V7, PSMA, and prostate-specific antigen (PSA) mRNA expression using the AdnaTest ProstateCancerPanel AR-V7 assay.

**Results:**

AR-V7 expression could be detected in 22Rv1 cells spiked into blood from healthy volunteers as well as in CTCs and plasma-derived exosomal cfRNA from patients with mCRPC by processing blood with the VTX-1 CTC isolation system followed by the AdnaTest ProstateCancerPanel AR-V7 assay. 94.7% of patient blood samples (18/19) had detectable AR expression in either CTCs or exosomal cfRNA (16 in CTCs, 12 in cfRNA). 15.8% of the 19 patient blood samples (3/19) were found to have AR-V7-positive (AR-V7+) CTCs, one of which was also AR-V7+ in the exosomal cfRNA analysis. 42.1% of patient blood samples (8/19) were found to be PSMA positive (PSMA+): 26.3% (5/19) were PSMA+ in the CTC analysis and 31.6% (6/19) were PSMA+ in the exosomal cfRNA analysis. Of those 8 PSMA+ samples, 2 had detectable PSMA only in CTCs, and 3 had detectable PSMA only in exosomal cfRNA.

**Conclusion:**

VTX-1 enables isolation of CTCs and plasma exosomes from a single blood draw and can be used for detecting AR-V7 and PSMA mRNA in both CTCs and cfRNA in patients with mCRPC and resistance to ARIs. This technology facilitates combining RNA measurements in CTCs and exosomal cfRNA for future studies to develop potentially clinically relevant cancer biomarker detection in blood.

**Supplementary Information:**

The online version contains supplementary material available at 10.1186/s12885-024-12139-3.

## Background

Prostate cancer (PC) is the second most common type of cancer in men worldwide and its growth is driven by androgens [1, 2]. Although androgen-deprivation therapy (medical castration) is the mainstay of treatment for metastatic disease, all patients eventually progress to metastatic castration-resistant prostate cancer (mCRPC). Effective treatment of mCRPC requires the addition of agents that include ARIs such as enzalutamide, abiraterone, apalutamide, darolutamide, or taxane chemotherapies such as docetaxel and cabazitaxel, and most recently, PSMA-directed therapy [3–6].

ARIs are used in first-line treatment for mCRPC and more recently also for castration-sensitive PC [7, 8]. However, about one third of the patients show primary resistance to abiraterone and enzalutamide treatment without blood PSA decline, and almost all initial responders develop secondary resistance over time [9, 10]. The presence of AR-V7, the most abundant splice variant, plays an important role in resistance to ARIs: AR-V7 lacks the androgen-binding site and remains constitutively active as a transcription factor, independent of androgen signaling [11]. The clinical importance of this finding was demonstrated by showing that AR-V7 is associated with resistance to abiraterone and enzalutamide and shorter overall survival in patients with mCRPC [12–21]. Thus, AR-V7 is both a prognostic and a predictive biomarker.

Liquid biopsies of CTCs can be used in patients with mCRPC to assess AR-V7 status [12–15, 17–21]. The detection of AR-V7 in the nucleus of CTCs (i.e., not in the cytoplasm) with the Oncotype DX AR-V7 Nucleus Detect test by Epic Sciences has been developed to guide treatment decisions for patients with mCRPC [13, 14, 17]. Alternatively, AR-V7 mRNA levels can be quantified with assays such as the AdnaTest ProstrateCancerDetect AR-V7 quantitative polymerase chain reaction (qPCR) assay by QIAGEN (AdnaDetect). The AdnaTest workflow includes two steps: [1] AdnaSelect enables the immunomagnetic enrichment of CTCs from peripheral blood using magnetic beads conjugated to antibodies against epithelial (such as epithelial cell adhesion molecule [EpCAM]) and tumor-associated antigens on the cell surface [2]. AdnaDetect, a qPCR-based assay, detects AR-V7 mRNA expression as well as AR-wildtype, PSMA, PSA, CD45 (white blood cell [WBC] marker) and glyceraldehyde 3-phosphate dehydrogenase (GAPDH; internal standard) in the enriched cell preparations [22].

Despite the presence of multiple antibodies in AdnaSelect to capture a broad range of CTCs, cell-marker based CTC enrichment requires prior knowledge of the markers of interest (e.g., EpCAM) to enable the capture and may miss the CTCs without the targeted antigens. Marker-independent CTC enrichment technologies are thus still needed to rapidly isolate a broader spectrum of CTCs in patients with PC [23]. Furthermore, previous studies have suggested the presence of AR-V7 transcripts not only in CTCs but also in exosomal cfRNA [24] and plasma cfRNA [25]. Thus, detecting AR-V7 in both CTCs and cfRNA might provide a more complete AR-V7 profile of the patient from a single blood draw. Given the recent approval of Lu177-PSMA targeted radiotherapy in patients with PSMA-positive mCRPC, we also report on PSMA detection in blood, as a PSMA blood test could contribute in the future to directing PSMA-targeted treatment selection and monitoring of PSMA-positive tumor burden in blood [26–33].

The VTX-1 Liquid Biopsy System (Vortex Biosciences) is a microfluidic label-free CTC isolation system that captures CTCs based on their physical characteristics instead of cell surface markers, with high CTC recovery and purity in a simple and fully automated process [34–36]. The blood sample can be depleted of the blood plasma first and then processed using the VTX-1 to recover the CTCs, thereby enabling the analysis of gene expression in both CTC and exosomal cfRNA from the same patient blood sample.

**Fig. 1:**
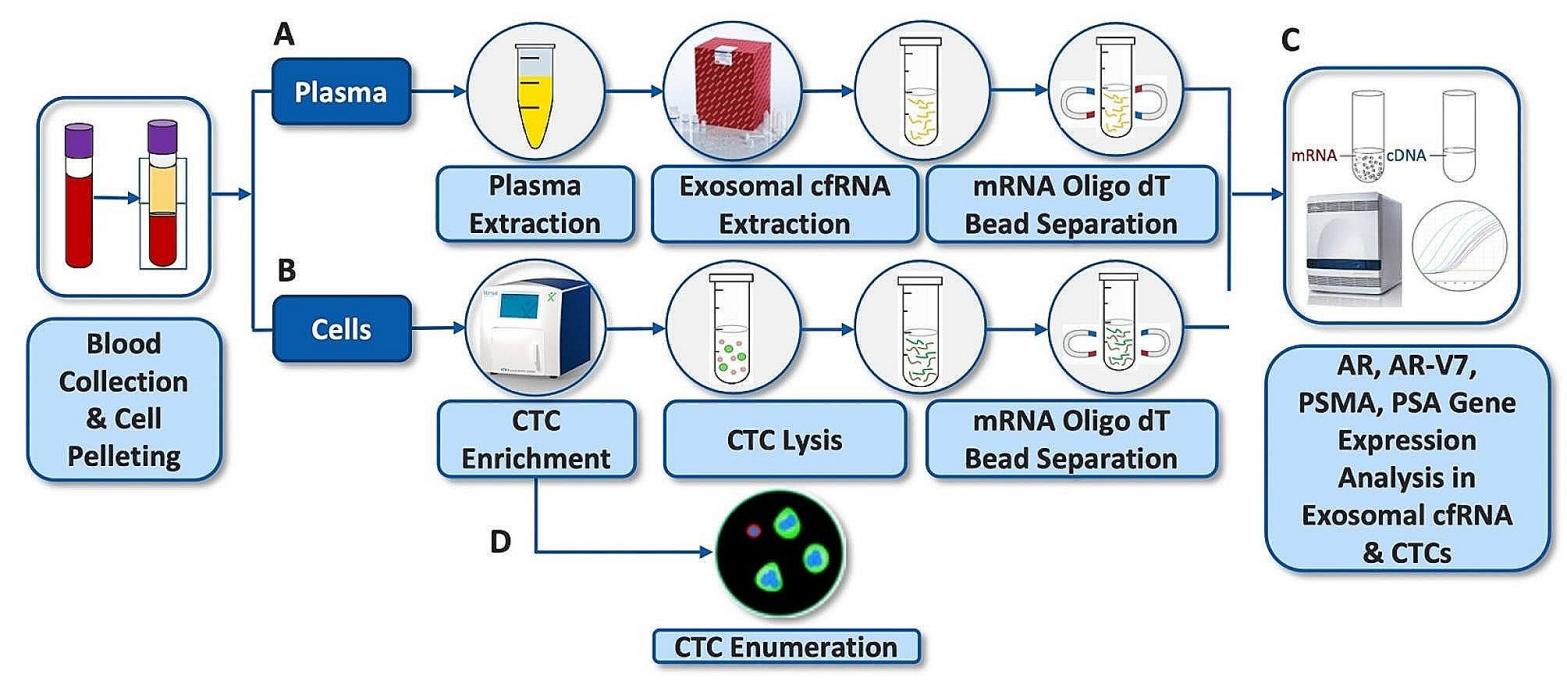
Workflow for AR, AR-V7, PSMA, and PSA AdnaTest applied to exosomal cfRNA and VTX-1-isolated CTCs. Blood was collected from patients with mCRPC and resistance to ARI inhibitors. Plasma was extracted from whole blood by centrifugation. (**A**) Exosomal cfRNA was extracted from plasma exosomes using exoRNeasy Serum Plasma Kit (QIAGEN). (**B**) CTCs were isolated from the plasma-depleted blood with VTX-1 and lysed using the Adna lysis/binding buffer. (**C**) CTC lysate and exosomal cfRNA were assayed for expression of AR, AR-V7, PSMA, and PSA mRNA with AdnaDetect. (**D**) A separate tube of whole blood was processed with VTX-1 in parallel for CTCs enumeration. mCRPC: metastatic castration-resistant prostate cancer; CTC: circulating tumor cell; mRNA: messenger RNA; AR: androgen receptor; AR-V7: AR splice variant 7; PSMA: prostate-specific membrane antigen; PSA: prostate-specific antigen; cfRNA: cell-free RNA

In this study, we evaluated the integration of VTX-1 with AdnaDetect into a seamless workflow (Fig. 1). We used the VTX-1 to isolate CTCs from the plasma-depleted blood (the plasma being then used for exosome extraction). Then, wild-type AR, AR-V7, PSMA, and PSA mRNA expression levels were assessed in CTCs and plasma exosomal cfRNA, using AdnaDetect. The workflow was then applied to patient samples to compare detectability of AR-V7 expression in CTCs and exosomal cfRNA and to evaluate whether there is potential synergy between these two liquid biopsies.

## Methods

### Cell lines

22Rv1 (human prostate cancer cell line, ATCC CCL-2505) and MCF7 (human breast cancer cell line, ATCC HTB-22) were both grown in RPMI-1640 medium (Gibco), supplemented with 10%(v/v) fetal bovine serum (HyClone) and 1%(v/v) Penicillin-Streptomycin (Corning). For MCF7 cells, human recombinant insulin (Life Technologies) was added to the medium at a final concentration of 0.01 mg/mL. Both cell lines were grown at 37° C with 5%(v/v) CO_2_ and tested for mycoplasma contamination before the experiments (Venor GeM, Sigma). For all experiments, cells were dissociated using TrypLE express (Gibco) at a confluency of 30–60%. The concentration of live cells per mL, viability, and average diameter were measured using Acridine Orange/Propidium Iodide (AOPI) solution with an automated cell counter (Nexcelom, Cellometer K2). Cell suspensions were serially diluted in complete medium to obtain the desired final concentrations. To obtain an accurate estimate of the cell number, the final dilution was stained with Calcein AM for 10 min at room temperature (RT), then 200 µL of each cell suspension were dispensed into a 96-well plate and manually counted using a 5x objective under brightfield and fluorescent illumination (Axio Observer Z1, Zeiss).

### Blood donor selection and blood collection

For combined VTX-1-AdnaDetect assay development and characterization, blood samples were obtained from healthy donors recruited at Stanford University School of Medicine with the donors’ written informed consent under a clinical study protocol approved by the Stanford Institutional Review Board (Stanford IRB #5630).

For combined VTX-1-AdnaDetect assay testing, blood samples were collected from each of 16 patients with mCRPC and rising PSA. Each patient was either currently taking an ARI or had previously received and progressed while receiving an ARI. Thirteen of the 16 patients had blood drawn at a single time point. Three of the 16 patients agreed to a repeat blood draw at a second time point. Therefore, a total of 19 blood samples from 16 patients were analyzed. All blood samples were obtained at Stanford Healthcare after written informed consent from the subjects under a clinical study protocol approved by the Stanford Institutional Review Board (Stanford IRB #12597). All blood samples were blinded to the investigators at Vortex Biosciences performing the blood processing, CTC isolation, and gene expression analyses. Patient clinical and demographic data are summarized in Supplementary Tables 1, 2, and 3.

All blood samples were collected by venipuncture in EDTA (for initial optimization of blood collection conditions) or ACD-A blood collection tubes (for initial optimization of blood collection conditions and for all patient samples) (all from BD) and gently inverted 10 times immediately after the draw. Blood samples were immediately transported to Vortex Biosciences at room temperature (RT) or cooled (4–10° C). Specifically, patient samples were surrounded by bubble wrap with a temperature logger, and transported in an insulated box with one frozen gel pack and three gel packs pre-chilled at 4° C. All samples were processed within 4 to 30 h.

### Blood processing and overall workflow

As controls for cell mixing, immunofluorescence staining, and gene expression analysis, WBCs were isolated from whole blood from healthy donors (see Sect. 'White blood cell preparation' below).

For cell spike-in experiments, a known number of 22Rv1 or MCF7 cells was spiked into the desired volume of blood from healthy donors and then processed with VTX-1 (see Sect. 'Blood processing with VTX-1 and CTC collection' below). Isolated cells were collected either for gene expression analysis (see Sect. 'Gene expression analysis of CTCs and cfRNA with the AdnaDetect assay' below) or for staining and enumeration to calculate the capture efficiency (see Sect. 'Immunofluorescence staining and cell enumeration' below).

Patient blood samples were processed following the workflow described in Fig. 1: Blood was collected in three 10 mL ACD-A blood collection tubes from each patient at each time point. From two of these tubes, 4 mL plasma each were extracted (see Sect. 'Plasma separation' below) and pooled, and 8 mL of pooled plasma were used to isolate exosomal cfRNA (see Sect. 'Exosomal cfRNA extraction' below). The removed plasma volume was immediately replaced with PBS and the blood was resuspended to constitute the plasma-depleted blood (PDB) for CTC isolation. Then, the PDB from each tube was pooled and 16 mL processed with VTX-1 to enrich the CTCs. Plasma exosomal cfRNA (see Sect. 'Exosomal cfRNA extraction' below) and collected CTCs (see Sect. 'Blood processing with VTX-1 and CTC collection' below) were analyzed for AR, AR-V7, PSMA, and PSA gene expression (see Sect. 'Gene expression analysis of CTCs and cfRNA with the AdnaDetect assay' below). From the third tube of blood, 8 mL of blood were processed with VTX-1 in parallel without prior plasma depletion to isolate CTCs for enumeration (see Sect. 'Immunofluorescence staining and cell enumeration' below).

### White blood cell preparation

1 mL of whole blood was mixed with 5 mL of Erythrocyte Lysis Buffer (Buffer EL, QIAGEN) and incubated on ice for 15 min, centrifuged at 400 *g* at 4° C for 10 min, and the supernatant removed. The pellet was resuspended in 2 mL of Buffer EL. After centrifugation (same conditions as before), the pellet was resuspended in 1 mL of complete RPMI-1640 medium. Final dilutions of the WBC suspension were stained with 1:200 of Hoechst nuclear stain (Molecular Probes) and 1 µL Calcein AM (Thermo Fisher) for 10 min at RT. Then, 200 µL of the suspension was dispensed into a well of a 96-well plate, imaged (5X objective, brightfield and fluorescence microscopy), and cells counted.

### Plasma separation

Each blood collection tube was gently inverted 10 times and normalized to 8 mL of blood (with the excess of blood being removed for volume consistency) and centrifuged at 1,300 *g* at 4° C for 10 min without brakes during deceleration to separate the blood into plasma, buffy coat, and blood cell pellet. Plasma was gently aspirated without disturbing the buffy coat and transferred to a 15 mL tube. The extracted plasma was then centrifuged at 3,700 *g* at 4° C for 15 min with slow deceleration. After centrifugation, the plasma supernatant was taken off without disturbing the cell pellet. Plasma from the two blood collection tubes from each time point was pooled, and the sample snap-frozen and stored at -80° C.

### Exosomal cfRNA extraction

Exosomal cfRNA was extracted from blood plasma using exoRNeasy Serum Plasma Kit (QIAGEN) according to the manufacturer’s protocol. Briefly, thawed plasma was passed through a 0.8 μm pore size syringe filter (Sartorius) to ensure removal of large particles, apoptotic bodies, cell debris and remaining cells. Exosomes were isolated from filtered plasma by affinity binding to an exoEasy spin column. Total RNA was isolated from column-bound exosomes by chloroform extraction and purified over a column (RNeasy MinElute, QIAGEN). Extracted exosomal cfRNA was finally eluted in 14 µL of water. 200 µL of Adna Lysis Buffer (QIAGEN) was added and the sample stored at -80° C until batched gene expression analysis with the AdnaDetect assay (QIAGEN).

### Blood processing with VTX-1 and CTC collection

Blood was processed using the VTX-1 Liquid Biopsy System (Vortex Biosciences) as described previously [34, 37]. For each run, a 4 mL aliquot of whole blood or PDB was diluted 10-fold with PBS and run through the VTX-1 microfluidic chip. For patient samples, a total of 16 mL of PDB were processed in 4 consecutive runs (for gene expression analysis) or 8 mL of whole blood were processed in 2 consecutive runs (for CTC enumeration). The VTX-1 was operated in ‘high-recovery mode’ with 3 cycles per run (optimized for maximum CTC yield): after the first and second cycle, the flow-through is automatically loaded into the VTX-1 again and processed for another cycle to isolate additional cancer cells that were not captured in the previous cycle. For spike-in experiments, the output from each cycle was analyzed separately. For patient samples, the isolated cells from all 3 cycles were pooled for analysis.

For gene expression analysis, the cells enriched with VTX-1 were collected in a 1.5 mL tube and centrifuged at 400 *g* at RT for 10 min. The bulk of the supernatant was carefully aspirated, leaving ∼ 50 µL of PBS. 200 µL of Adna Lysis Buffer (QIAGEN) were added and the sample stored at -80° C until batched gene expression analysis.

For cell immunofluorescence staining and enumeration, the cells enriched with VTX-1 were collected in an 8-well strip, fixed at RT with 4% paraformaldehyde (Electron Microscopy Sciences) for 10 min, and stored at 4° C until staining.

### Gene expression analysis of CTCs and cfRNA with the AdnaDetect assay

The AdnaDetect assay was used according to the manufacturer’s protocol:

*Oligo-dT based mRNA isolation and reverse transcription.* The required amount of Oligo-dT bead suspension was washed with lysis-binding buffer, added to each of the CTC and cfRNA lysates, and incubated at RT with gentle mixing to allow the binding of mRNA to the beads. The mRNA-bead complexes were then washed with buffer, resuspended in RNase-free water, and immediately used for reverse transcription into cDNA using the Sensiscript RT Kit (QIAGEN) and maximum volume of RNA as input. A negative control was included for the reverse transcription.

*Pre-amplification and qPCR.* The cDNA samples were pre-amplified using the Multiplex PCR Plus Kit (QIAGEN) for 12 cycles. The pre-amplified cDNA was diluted 1:10 before performing the qPCR analysis with the AdnaDetect assay. QuantiNova SYBR Green RT-PCR kit (QIAGEN) was used for the qPCR, with a positive control template and an internal control primer and template. The gene panel consisted of AR, AR-V7, PSMA, PSA, CD45 (leukocyte marker), and GAPDH (internal standard). 35 or 40 cycles of final qPCR were performed for each sample. Samples were run in duplicates. Per the manufacturer of the AdnaDetect test, with up to 18 cycles pre-amplification and up to 35 cycles final amplification, any signal in the assay has a specificity of > 90%, hence for this study, signals with Ct values below 35 were considered positive and specific for the assayed marker [22].

### Immunofluorescence staining and cell enumeration

Fixed cells were permeabilized with a 1:1 solution of 0.4% (v/v) Triton X-100 (Research Products International Corp) and 10% Goat Serum (Invitrogen) at RT for 7 min, blocked with goat serum at RT for 30 min, and labeled with 4′,6-diamidino-2-phenylindole (DAPI; Life Technologies), phycoerythrin-anti CD45 antibody (clone HI30, BD Biosciences), and a cocktail of antibodies targeting cytokeratins (CK) (Alexa Fluor 488-anti pan-CK, clones AE1/AE3 [eBioscience]; FITC-anti CK, clone CK3-6H5 [Miltenyi Biotec]) at RT for 1 h. The cells were then imaged by immunofluorescence microscopy at 10x magnification (Axio Observer Z1, Zeiss) and identified and enumerated using the Zen2 software (Zeiss): (i) For spike-in experiments, MCF7 or 22Rv1 cells were identified as DAPI+/CK+/CD45- or WBCs as DAPI+/CK-/CD45+. *Capture efficiency* was defined as the number of cancer cells collected after VTX-1 processing divided by the number of cancer cells that were initially spiked into the blood sample (expressed as percentage). *Capture purity* was calculated as the number of cancer cells collected after VTX-1 processing divided by the total number of cells collected (i.e., cancer cells plus WBCs, expressed as percentage). (ii) For patient blood samples, collected cells were identified as CTCs or WBCs by immunostaining of CK, CD45, and DNA stained with DAPI, and considering nucleus size and nucleus-to-cytoplasm (N:C) ratio, relying on criteria defined previously [35]. CTCs were defined as either DAPI+/CK+/CD45- cells or DAPI+/CK-/CD45- cells with a large nucleus (diameter > 9 μm) and high N:C ratio (> 0.8). Most WBCs were DAPI+/CK-/CD45+. Several WBCs stained double-positive for CK and CD45 and were identified previously as activated granulocytes [35].

### Oncotype DX AR-V7 nucleus detect test

Three of the 19 blood samples (from 2 patients with mCRPC) had a corresponding clinical Oncotype DX AR-V7 Nucleus Detect Test result (Epic Sciences) performed as part of the patients’ routine clinical care.

## Results

### Characterization of the combined VTX-1-AdnaDetect workflow

#### Characterization of the workflow using pure cell lines

**Fig. 2:**
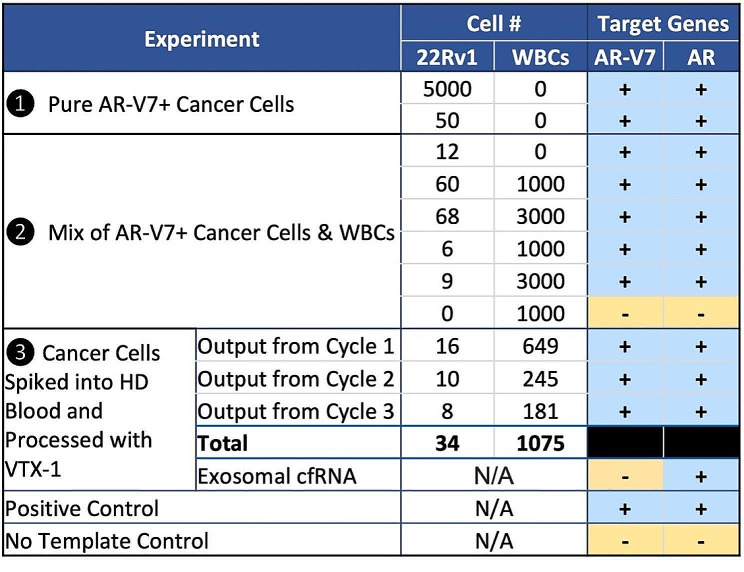
Characterization of the combined VTX-1-AdnaDetect workflow with cell lines. ❶ The indicated number of 22Rv1 cells was analyzed with AdnaDetect. ❷ Different quantities of 22Rv1 cells (12, 60, 68, 6, 9, 0) were mixed with a defined number of WBCs (0, 1000, 3000, 1000, 3000, 1000 cells, respectively) and analyzed with AdnaDetect. ❸ Fifty 22Rv1 cells were spiked into 4 mL blood from healthy donors, plasma depleted, and run for 3 cycles with the VTX-1, then the output from each cycle analyzed for AR-V7 and AR expression with AdnaDetect. AR: androgen receptor; AR-V7: AR splice variant 7; WBC: white blood cell; HD: healthy donor

To characterize the VTX-1-AdnaDetect workflow, we focused on AR and AR-V7. We first tested 22Rv1 cells, a human prostate cancer cell line shown to have high AR-V7 gene expression [38]. The performance of the AdnaDetect assay for AR, AR-V7, PSMA, and PSA has been published previously [22]. 5,000 and 50 22Rv1 cells were assayed with the AdnaDetect test, confirming detectable AR and AR-V7 expression (Fig. 2, ❶). All Ct values for the amplified transcripts are provided in Supplementary Table 4.

#### Characterization of the cell workflow using mixtures of 22Rv1 prostate cancer cells and purified WBCs

To confirm the detectability of AR-V7 and AR mRNA with the AdnaDetect assay in VTX-1-isolated cells, different cell mixtures of 22Rv1 prostate cancer cells and purified WBCs were prepared to mimic a typical VTX-1 output and analyzed for AR-V7 and AR gene expression: 0 to 68 22Rv1 cells were mixed with 0, 1,000, or 3,000 WBCs (Fig. 2, ❷). These numbers were based on previous data where the number of CTCs in patient blood samples processed with VTX-1 ranged from 0 to > 100, while the number of WBCs is on average lower than 100 per mL of blood per VTX-1 cycle [36, 37]. This corresponds to a total of ∼ 3,000 WBCs from 10 mL of blood after 3 cycles of processing in VTX-1 high-recovery mode. AR-V7 and AR were successfully detected in as little as 12 pure cancer cells, 6 cancer cells mixed with 1,000 WBCs (0.6% purity), and 9 cancer cells mixed with 3,000 WBCs (0.3% purity). No AR-V7 or AR signal was detected in the WBC-only sample. All Ct values for the amplified transcripts are provided in Supplementary Table 4.

#### Characterization of the combined VTX-1-AdnaDetect workflow with cancer cells spiked into whole blood

To mimic patient blood sample processing with the VTX-1, fifty 22Rv1 cells were spiked into 4 mL of blood from healthy donors that was collected in ACD-A tubes and stored at 4˚ C for ∼ 4 h. These conditions were selected based on recommendations of the QIAGEN AdnaDetect manual and our own benchmarking experiments (Supplementary Fig. 1). Spike-in blood samples were then depleted of plasma and processed with the VTX-1 for 3 cycles. The cell output was collected separately from each cycle and analyzed with the AdnaDetect assay (Fig. 2, ❸). A duplicate sample was processed with VTX-1 in parallel for cell enumeration. AR-V7 and AR were well detected in the output from each cycle, i.e., in 16, 10 and eight 22Rv1 cells isolated from cycles 1, 2, and 3, respectively (68% overall capture efficiency). In parallel, the plasma was separated ∼ 4 h after the cancer cells were spiked into the blood. The exosomal cfRNA was extracted and analyzed with AdnaDetect in parallel to the samples processed with VTX-1 (Fig. 2, ❸). AR, but not AR-V7 was detected in the plasma exosomal cfRNA. This suggests that there is no significant apoptosis or EV excretion from spiked-in cancer cells within 4 h of blood storage at 4˚ C. These results confirm the validity of the entire workflow, i.e., blood collection in ACD-A tubes, storage at 4˚ C, plasma depletion and exosomal cfRNA extraction, VTX-1 processing of plasma-depleted blood, and AR-V7/AR expression analysis with AdnaDetect in both CTCs and plasma exosomal cfRNA. All Ct values for the amplified transcripts are provided in Supplementary Table 4.

### Isolation and enumeration of CTCs from blood samples from patients with mCRPC

**Fig. 3:**
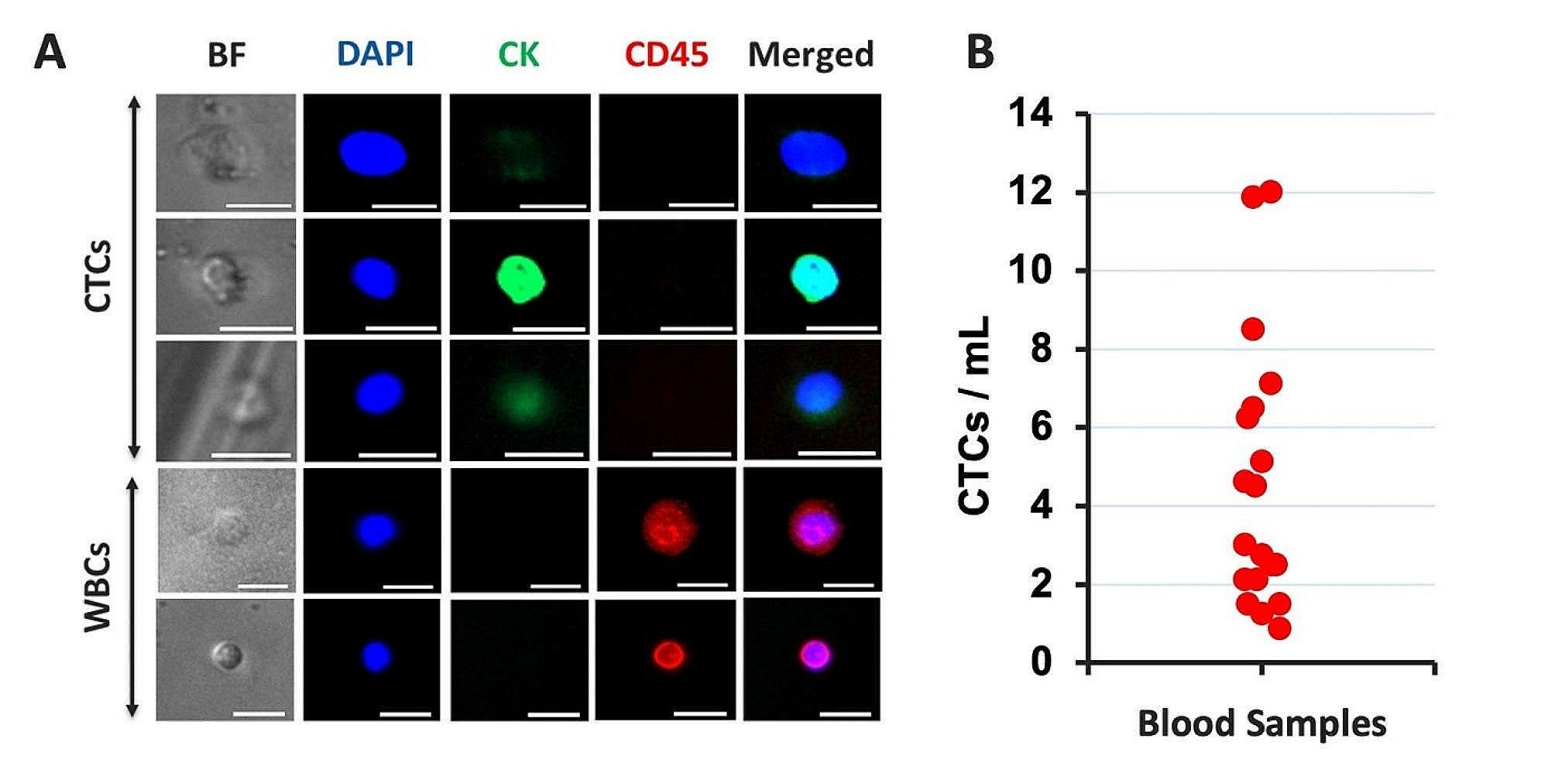
Heterogeneity of marker expression in CTCs in blood samples from patients with mCRPC and resistance to ARI therapy. (**A**) Bright-field (BF) and immunofluorescence images of CTCs and WBCs isolated with VTX-1 from patient blood samples immuno-stained for DNA (DAPI, blue), cytokeratins (CK, green), and CD45 (red). Scale bar: 20 μm. (**B**) Number of CTCs per mL of blood. mCRPC: metastatic castration-resistant prostate cancer; ARI: androgen receptor inhibitor; CTCs: circulating tumor cells; WBCs: white blood cells

In order to enumerate CTCs from each donor, blood from 16 patients with mCRPC and therapeutic resistance to ARIs was processed with VTX-1. Collected cells from 8 mL of blood were fixed, stained, and imaged. Figure 3A presents the range of marker expression in CTCs (either DAPI+/CK+/CD45- or DAPI+/CK-/CD45- plus nuclear diameter > 9 μm and nucleus-to-cytoplasm ratio > 0.8) and WBCs (DAPI+/CK-/CD45+) according to criteria described previously [35]. Supplementary Table 5 provides a break-down of CTC numbers by CK+/CK- staining and additionally by VIM+/VIM- staining where available. Figure 3B shows the number of identified CTCs per mL of blood for the cohort of patients (range: 0.88 to 12 CTCs/mL, corresponding to a total of 7 to 96 CTCs in 8 mL of blood).

### Gene expression analysis in CTCs and plasma exosomal cfRNA from patients with mCRPC and ARI resistance using the combined VTX-1-AdnaDetect workflow

Blood was sampled from 16 patients with mCRPC. Each of these patients had experienced clinical progression on ARI therapy. For the AdnaDetect assay, CTCs and plasma exosomal cfRNA from the 16 patients were extracted and prepared to detect AR, AR-V7, PSMA, and PSA expression using the combined VTX-1-AdnaDetect workflow described and characterized above. The results were analyzed as per the AdnaTest manual [22].

A few patients underwent a second serial blood draw, resulting in analysis of a total of 19 blood samples from 16 patients, as summarized in Table 1 (see Supplementary Table 6 for Ct values of the amplified genes). 94.7% of patient blood samples (18/19) had detectable AR expression in either CTCs or exosomal cfRNA (16 in CTCs, 12 in cfRNA). In 10 of these 18 AR-positive samples, AR was detected in both CTCs and exosomal cfRNA, in 6 samples AR was detected only in CTCs, and in 2 samples AR was detected only in exosomal cfRNA. 15.8% of patient blood samples (3/19) were found to have AR-V7-positive (AR-V7+) CTCs, one of which was also AR-V7+ in the exosomal cfRNA analysis. Two of these AR-V7+ samples corresponded to two different time points from the same patient.

Of the 19 patient blood samples, 42.1% (*n* = 8) were found to be PSMA positive (PSMA+): 26.3% (*n* = 5) were PSMA+ in the CTC analysis and 31.6% (*n* = 6) were PSMA+ in the exosomal cfRNA analysis. Of these 8 PSMA+ samples, 3 had detectable PSMA in both CTCs and exosomal cfRNA, 2 had detectable PSMA only in CTCs, and 3 had detectable PSMA only in exosomal cfRNA. Lastly, PSA was detected in 31.6% of samples (6/19): 3 samples in both CTCs and exosomal cfRNA, and 3 samples only in exosomal cfRNA.

The 3 samples that were found to express AR-V7 were also positive for PSMA and/or PSA (though the sample from patient #6 only had detectable PSMA, not PSA, and only in exosomal cfRNA, not in CTCs). Nevertheless, overall, this supports that the cells with AR-V7 signal are indeed CTCs. GAPDH and CD45 were also included as controls in the gene panel, with the corresponding Ct values listed in Supplementary Table 6.

**Table 1:** VTX-1-AdnaDetect test results of CTC and exosomal cfRNA analysis in 19 blood samples from 16 patients with mCRPC and resistance to ARI therapy. mCRPC: metastatic castration-resistant prostate cancer; AR: androgen receptor; AR-V7: AR splice variant 7; PSMA: prostate-specific membrane antigen; PSA: prostate-specific antigen; CTCs: circulating tumor cells; cfRNA: cell-free RNA.

Patient ID	Sample #	AR		AR-V7		PSMA		PSA	
		CTC	cfRNA	CTC	cfRNA	CTC	cfRNA	CTC	cfRNA
1	1	+	+	+	-	+	+	+	+
	**2**	**+**	**+**	**+**	**+**	**+**	**+**	**+**	**+**
**2**	**1**	**+**	**+**	**-**	**-**	**+**	**-**	**-**	**-**
	**2**	**+**	**-**	**-**	**-**	**-**	**-**	**-**	**-**
**3**	**1**	**+**	**+**	**-**	**-**	**-**	**-**	**-**	**+**
**4**	**1**	**+**	**+**	**-**	**-**	**+**	**+**	**+**	**+**
**5**	**1**	**+**	**-**	**-**	**-**	**-**	**-**	**-**	**-**
**6**	**1**	**+**	**-**	**+**	**-**	**-**	**+**	**-**	**-**
**7**	**1**	**+**	**-**	**-**	**-**	**-**	**+**	**-**	**-**
**8**	**1**	**-**	**+**	**-**	**-**	**-**	**-**	**-**	**-**
**9**	**1**	**+**	**+**	**-**	**-**	**+**	**-**	**-**	**-**
	**2**	**+**	**+**	**-**	**-**	**-**	**+**	**-**	**-**
**10**	**1**	**+**	**+**	**-**	**-**	**-**	**-**	**-**	**-**
**11**	**1**	**+**	**+**	**-**	**-**	**-**	**-**	**-**	**+**
**12**	**1**	**+**	**+**	**-**	**-**	**-**	**-**	**-**	**-**
**13**	**1**	**-**	**-**	**-**	**-**	**-**	**-**	**-**	**-**
**14**	**1**	**-**	**+**	**-**	**-**	**-**	**-**	**-**	**-**
**15**	**1**	**+**	**-**	**-**	**-**	**-**	**-**	**-**	**-**
**16**	**1**	**+**	**-**	**-**	**-**	**-**	**-**	**-**	**+**

### Longitudinal patient cases

**Fig. 4:**
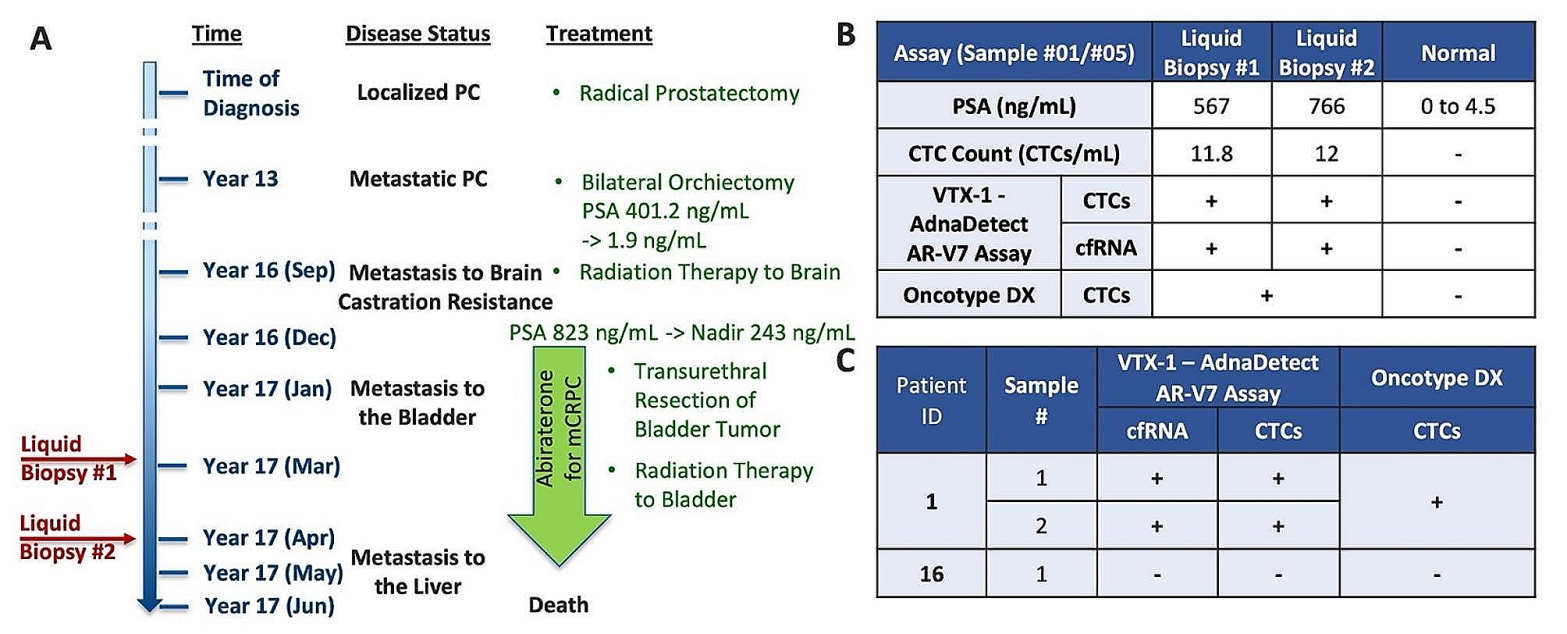
Patient case studies. (**A**) Clinical course of patient #1 and timing of blood sampling over the course of his treatment. (**B**) Liquid biopsies and blood markers at two time points: PSA value, CTC numbers, AR-V7 results from both the VTX-1-AdnaDetect assay and the Oncotype DX AR-V7 Nucleus Detect test. (**C**) Side-by-side comparison of the AR-V7 results for three blood samples from 2 patients between the VTX-1-AdnaDetect assay and the Oncotype DX AR-V7 Nucleus Detect test. PC: prostate cancer; PSA: prostate-specific antigen; CTC: circulating tumor cell; AR-V7: androgen receptor splice variant 7; cfRNA: cell-free RNA.

Amongst this cohort of patients, patient #1 allowed for in-depth clinical correlation of AR-V7 status (Fig. 4A). This patient was initially diagnosed and treated for localized prostate cancer with radical prostatectomy. Thirteen years after diagnosis, he developed metastatic prostate cancer in bones (PSA: 401.2 ng/mL), treated with surgical castration (bilateral orchiectomy). He subsequently developed castration-resistant prostate cancer with metastasis to the brain (PSA: 823 ng/mL). He received focal radiation to the brain lesions and initiated abiraterone treatment, with a decrease in PSA and a nadir PSA value of 243 ng/mL. He became resistant to abiraterone and developed progressive disease metastatic to the bladder, and despite radiation to the bladder, PSA rose to 567 ng/mL in March of year 17 after diagnosis when the first blood sample was taken. A month later, when the second blood sample was drawn, his PSA had risen further to 766 ng/mL. AR-V7 was detected at both time points in both CTCs and exosomal cfRNA using the VTX-1-AdnaDetect assay (Fig. 4B). Consistently, the CTC count was high (11.8 CTCs/mL and 12 CTCs/mL, respectively) confirming the reproducibility of the CTC counts and the AR-V7 analysis. Clinically, the AR-V7-positive results and the high CTC count were concordant with lack of response of the patient to abiraterone and progression of disease.

Similarly, patient #6 presented an interesting clinical course (Supplementary Fig. 2). This patient was diagnosed with *de novo* metastatic CRPC and high tumor burden. After initial clinical response to chemical castration, he developed castration resistance and was also resistant to subsequent treatment with abiraterone. At that time, he was tested positive for AR-V7 in CTCs in this study with the combined VTX-1-AdnaDetect assay (but not in cfRNA) and for PSMA in cfRNA (but not in CTCs). He then received radium-223 treatment followed by enzalutamide, to which he responded. Potential interpretations could be: the AR-V7 level, if accurate, was not sufficient to confer resistance to enzalutamide; or the AR-V7 expression had reversed to wildtype AR expression. The patient then was treated with cabazitaxel with moderate biochemical response, followed by the immune checkpoint inhibitor pembrolizumab (for high tumor mutational burden), to which he was resistant. After a positive PSMA-PET scan, the patient received PSMA-directed Lu-177 therapy with a marked improvement to his PSA values. Of note, although the blood sampling for PSMA preceded the PSMA scan and PSMA-directed Lu-177 treatment by more than three years, PSMA expression detected in exosomal cfRNA with the VTX-1-AdnaDetect assay correlated with a positive PSMA-PET scan and response to PSMA-targeted Lu-177 therapy.

### Comparison of results from VTX-1-AdnaDetect assay and Oncotype DX AR-V7 nucleus detect test

To compare our combined VTX-1-AdnaDetect workflow to the Oncotype DX AR-V7 Nucleus Detect test from Epic Sciences, three blood samples from 2 patients were collected and analyzed with the VTX-1-AdnaDetect assay at the time or within two weeks of their clinical Oncotype DX AR-V7 Nucleus Detect test. For both patients, each of the blood samples obtained concordant results with respect to AR-V7 detection in the VTX-1-AdnaDetect assay and in the Oncotype DX AR-V7 test (Fig. 4C). These blood samples were obtained before PSMA imaging became available as standard of care, so no PSMA imaging was available within the 12 months before or after the blood draw for comparison.

## Discussion

In this study, we tested a workflow combining a CTC isolation step using the VTX-1 Liquid Biopsy System (Vortex Biosciences) with the AdnaTest ProstateCancerPanel AR-V7 (QIAGEN) to detect expression of wild-type AR, AR-V7, PSMA, and PSA in both CTCs and plasma exosomal cfRNA from patients with mCRPC and therapeutic resistance to ARIs.

So far, the VTX-1 system has been used for the isolation of CTCs from breast, colorectal, non-small cell lung, and prostate cancer, for downstream CTC enumeration, Sanger sequencing, targeted next-generation sequencing, or single-cell Western blotting [23, 39–41]. This is the first application of a multiplexed downstream qPCR-based assay. AdnaTest, developed by QIAGEN, includes two steps [22]. The first step (AdnaSelect) is the capture of CTCs using immunomagnetic beads labeled with a mixture of different antibodies against EpCAM and markers of epithelial-to-mesenchymal transition (EMT) to ensure the capture of a broad range of CTCs. The second step (AdnaDetect) consists of the mRNA expression analysis using multiplexed reverse-transcription qPCR (RT-qPCR) primers. VTX-1 technology, instead, captures CTCs based on their physical characteristics using a microfluidic, label-free, and fully automated CTC isolation system with high CTC recovery and purity [35, 36]. The hybrid workflow described here, combining VTX-1 CTC isolation and qPCR profiling with AdnaDetect, was successfully validated and applied to patient samples, highlighting both the flexibility of VTX-1 output collection and the ease of use of the AdnaDetect assay.

Notably, the cells collected by the VTX-1 were intact, making them ideal for RNA analysis, with a typical overall cellular content of about 1,000–3,000 cells from one tube of blood. The samples isolated with VTX-1 are a mixture of CTCs, WBCs, and potentially other rare cell types. As per criteria described previously [35], CTCs were defined and identified based on lack of CD45 expression plus either expression of cytokeratins or nuclear diameter > 9 μm and N:C ratio > 0.8. We acknowledge that not all these cells might be CTCs but some could represent other rare cell types in blood. Nevertheless, samples processed with VTX-1 allowed us to detect AR with AdnaDetect in almost all cases, many of which were also positive for PSMA, indicating the presence of prostate cancer CTCs at sufficient purity.

Different approaches to interrogate AR-V7 have been studied: at the mRNA level using qPCR or digital droplet PCR (ddPCR), and at the protein expression level using staining like the Epic Biosciences Oncotype DX AR-V7 Nucleus Detect Test [15, 24, 42, 43]. In this study, we used a combined VTX-1-AdnaDetect workflow to quantify AR, AR-V7, PSMA, and PSA mRNA levels in patients with mCRPC by RT-qPCR. This combined workflow was compared to the Oncotype DX AR-V7 Nucleus Detect assay in two patients with 100% concordance. Determining the technical performance of the combined VTX-1-AdnaDetect assay in detail and further side-by-side comparisons will be needed to assess if its sensitivity and specificity are comparable to other commercial tests. Interestingly, in most published studies, detectable AR-V7 predicted poor outcomes of ARI therapy, regardless of AR-V7 detection method (Johns Hopkins University [JHU] AR-V7 mRNA assay or Oncotype DX AR-V7 protein assay) [14]. Armstrong et al. showed that these two assays were independently associated with shorter progression-free survival (PFS) and overall survival (OS), and there was 82% concordance between the two assays. For the JHU assay, median radiographic PFS and median OS were 3.1 and 10.8 months for AR-V7 + disease versus 6.9 and 27.2 months for AR-V7- disease. For Oncotype DX, the median radiographic PFS and median OS were 3.1 and 8.4 months for AR-V7 + disease versus 6.1 and 20.3 months for AR-V7- disease [18]. Some studies showed that AR-V7 protein localized to the cell nucleus does not always correlate with detectable AR-V7 mRNA but is more predictive of ARI therapy outcomes [14]. Nevertheless, the mRNA assay is decentralized and more accessible to the scientific community, while being also simpler and faster, indicating that the mRNA assay could provide a significant advantage for patient care.

In this study, we simultaneously analyzed AR, AR-V7, PSMA, and PSA in the CTCs and plasma exosomal cfRNA from the same tube of blood. This demonstrates that the VTX-1 system is compatible with a ‘total liquid biopsy.’ Even though CTCs are frequently used to interrogate AR-V7, plasma exosomal cfRNA is valid for AR-V7 gene expression studies [24].

In our study, 15.8% of blood samples (3/19) were found to be AR-V7+ in the CTC analysis, one of which was also AR-V7+ in the exosomal cfRNA analysis. All samples that tested positive for AR-V7 were also positive for PSA and PSMA, indicating that the AR-V7 signal may indeed come from prostate cancer cells. The 16 patients sampled had all progressed on an ARI, and a higher AR-V7+ rate was expected. One potential confounding factor is that, at the time of blood draw, seven of 16 patients had already switched to a different, non-ARI therapy (Supplementary Table 1). Although these seven patients had previously been resistant to ARI and had rising PSA on their current treatment, AR-V7 expression has been shown to be reversible [44]. Therefore, it is possible that the window of detectable AR-V7 expression was missed in some patients. Moreover, mechanisms of resistance to ARIs other than AR-V7 have been described, for example, somatic mutations in the androgen receptor gene or activation of alternative signaling pathways [45]. In line with these possibilities, seven patients in our cohort went on to receive a subsequent ARI at some point after their blood draw (with or without other treatments in between the two ARIs). Of these seven, four had decreases in PSA in response to the subsequent ARI (Supplementary Table 3). Together, this might explain the lower AR-V7 positive rate in our study compared to other studies: 49% (18 of 37 patients with detectable CTCs) in CTCs isolated and analyzed by RT-qPCR with the AdnaTest ProstateCancerPanel kit and 28.8% in CTCs isolated with the CellSearch CTC Test and analyzed by RT-qPCR [19, 46]. An expanded study of blood and prostate cancer tissue samples from the same patients will be needed to distinguish between true- and false-negative results of our combined VTX-1-AdnaDetect assay and will need to include patients with lower tumor burden than in this study cohort, which was enriched for patients with high PSA.

For the 18 AR+ blood samples, the discordance in AR positivity was 44.4% (8/18), as they had detectable AR in either CTCs or exosomal cfRNA, but not in both. Similarly, the discordances were 66.7% for AR-V7+ samples (2/3), 62.5% for PSMA+ samples (5/8) and 50% of PSA+ samples (3/6) between CTCs and exosomal cfRNA. This finding of a high discordance of RNA profiles between CTCs and exosomes is a known phenomenon: Del Re et al. were the first to report AR-V7 detection in blood plasma exosomes using ddPCR in 36 patients with mCRPC that were previously treated with enzalutamide or abiraterone and found a markedly prolonged progression-free survival in AR-V7- patients compared to previous reports from CTC analysis [24]. The investigators attributed this to a potentially higher percentage of false-negatives when analyzing CTCs, thereby claiming that assays in exosomal cfRNA might be more sensitive to detect AR-V7. Nimir et al., however, directly compared AR-V7 expression in CTCs to exosomal cfRNA in 16 patients with PC (9 of which with CRPC) and found that CTCs provide a sensitivity advantage over exosomes (7 samples AR-V7+ in CTCs, 2 of these also in exosomal cfRNA, discordance 71.4%; [47]). Similarly, Keup and colleagues determined a positive concordance of only 5.2% for 17 different RNA transcripts between CTCs and extracellular vesicles in patients with metastatic breast cancer [48]. In our study, utilizing the same methodology, the concordance for 3 RNA profiles (AR, PSMA, and PSA) between CTCs and exosomes for positive signals in both fractions was low at 33%. Interestingly, serial sampling obtained from the same patients (patients #2 and #9) revealed a dynamic appearance or disappearance of PSMA mRNA signals in CTCs and exosomes. This dynamic pattern of varying detectability of mRNA in CTCs and extracellular vesicles has also been reported in 27 patients with metastatic breast cancer who had sampling at three different time points [49]. The investigators have suggested that the detectability of mRNA in CTCs and extracellular vesicles correlates with disease progression and treatment success. Due to the dynamic nature of CTCs and extracellular vesicles, it is possible that measuring both CTCs and cfRNA together might better detect AR-V7 and PSMA than either one alone, especially when signals are close to the limit of detection, and will need to be investigated further in larger cohorts.

We were able to detect PSMA in CTCs and cfRNA. Because the majority of blood draws occurred prior to routine availability of PSMA-PET scans, only three patients received PSMA-PET scans subsequent to their blood samples as part of clinical trials (Supplementary Table 3). Notably, patient #6 who was PSMA+ in exosomal cfRNA, had a positive PSMA-PET scan several years after his blood draw and had an excellent partial response to Lutetium-177 (Lu-177) PSMA therapy, with an 83% reduction of PSA (baseline to nadir, Supplementary Fig. 2). The remaining patients that received PSMA-PET scans did not receive Lu-177 PSMA therapy. While CTCs and exosomal cfRNA did not reveal PSMA positivity for all patients who had positive scans, future studies can investigate whether detectable PSMA mRNA from exosomes or CTCs correlates to response to Lu-177 therapy.

Our study had several important limitations. Our sample size was small; a larger patient cohort, sampled over the course of their disease, will facilitate drawing more definitive conclusions about the clinical utility of our combined VTX-1-AdnaDetect assay and will be needed to determine if a combination of CTC and exosomal cfRNA analysis is more indicative of the prostate cancer status of a patient than either analysis alone. Another limitation was a heterogeneous patient population, with broad patient inclusion criteria that were not limited to documented detectable AR-V7 in CTCs or exosomes or detectable PSMA on PET/CT, nor a specific treatment regimen, preselected number of prior lines of therapy, extent of disease, or consistent timing of blood sampling after developing resistance to an ARI. Future studies in a larger patient population would allow us to investigate well-defined subsets of patients. An additional limitation is that we included only a select number of target genes in the assay instead of a larger panel of potentially relevant targets. Most importantly, there is currently no “gold standard” in the measurement of AR-V7 levels in CTCs or exosomes, against which we could validate our combined VTX-1-AdnaDetect assay.

Despite these limitations of our study, we did identify a patient with elevated numbers of CTCs and detectable AR-V7 in two consecutive blood draws, one month apart. This result showed consistency of the overall assay and suggested that more data points per patient may provide more valuable information on a patient’s AR-V7 status and its correlation with the number of CTCs and the PSA level throughout the course of the treatment.

## Conclusion

In summary, this research study introduced a workflow combining the VTX-1 Liquid Biopsy System for the label-free isolation of CTCs with the AdnaTest ProstateCancerPanel AR-V7 for the assessment of AR, AR-V7, PSMA, and PSA expression in both CTCs and plasma-derived exosomal cfRNA from the same tube of blood. Using both CTCs and exosomal cfRNA provides more material for a potentially more effective and sensitive AR-V7 assessment. For two patients, a side-by-side comparison of this combined workflow with the Oncotype DX AR-V7 Nucleus Detect test provided concordant results. This approach demonstrates that it is possible to detect AR-V7 and PSMA from a single blood sample. A larger and more homogenous cohort will be needed to understand potential clinical relevance of this combined workflow.

### Electronic supplementary material

Below is the link to the electronic supplementary material.

Supplementary Material 1

## Data Availability

All analytic data is made available in the Supplement to this publication. Additional data and materials are made available to the scientific community as needed upon request sent to any of the corresponding authors.

## Abbreviations

ACD-A: Acid-Citrate-Dextrose A
AR: Androgen Receptor
ARI: Androgen Receptor Inhibitor
AR-V7: Androgen Receptor Splice Variant 7
ATCC: American Type Culture Collection
CD45: Cluster of Differentiation 45
cDNA: Complementary DNA
cfRNA: Cell-free RNA
CK: Cytokeratin
Ct: Cycle Threshold
CTC: Circulating Tumor Cell
DAPI: 4′,6-Diamidino-2-Phenylindole
DNA: Desoxyribonucleic Acid
EDTA: Ethylenediamine Tetraacetic Acid
EMT: Epithelial-to-mesenchymal Transition
EpCAM: Epithelial Cell Adhesion Molecule
GAPDH: Glyceraldehyde 3-Phosphate Dehydrogenase
IRB: Institutional Review Board
mCRPC: Metastatic Castration-resistant Prostate Cancer
mRNA: Messenger RNA
PC: Prostate Cancer
PCR: Polymerase Chain Reaction
PDB: Plasma-depleted Blood
PET: Positron Emission Tomography
PSA: Prostate-specific Antigen
PSMA: Prostate-specific Membrane Antigen
qPCR: Quantitative PCR
RNA: Ribonucleic Acid
RT: Room Temperature
RT-PCR: Reverse Transcription-PCR
VIM: Vimentin
WBC: White Blood Cell
