# Supplementary material for: Detecting androgen receptor (AR), AR variant 7 (AR-V7), prostate-specific membrane antigen (PSMA), and prostate-specific antigen (PSA) gene expression in CTCs and plasma exosome-derived cfRNA in patients with metastatic castration-resistant prostate cancer (mCRPC) by integrating the VTX-1 CTC isolation system with the QIAGEN AdnaTest
Source: BMC Cancer. 2024 Apr 16;24:482. doi: 10.1186/s12885-024-12139-3 (PMC11022466; doi:10.1186/s12885-024-12139-3)
Supplement: Supplementary file 1 — Supplementary Material 1 [file 12885_2024_12139_MOESM1_ESM.docx]

**SUPPLEMENTARY FIGURES AND TABLES**

| **Patient ID** | **Sample #** | **Age**  **(in years)** | **PSA at**  **Time of Blood Draw**  **(in ng/mL)** | **PSA Doubling Time Prior to Blood Draw**  **(in months)** | **Sites of**  **Metastatic Disease** | **Disease Volume** | **Treatment Status** |
| --- | --- | --- | --- | --- | --- | --- | --- |
| **1** | **1** | **67** | **567.0** | **1.4** | **Liver, Bone** | **High** | ***Previous:***  **prostatectomy, focal radiation, orchiectomy**  ***Current (with rising PSA):***  **leuprolide, abiraterone** |
|  | **2** | **67** | **766.0** | **1.4** | **Liver, Bone** | **High** | ***Previous:***  **prostatectomy, focal radiation, orchiectomy**  ***Current (with rising PSA):***  **abiraterone** |
| **2** | **1** | **72** | **9.8** | **3.5** | **Bone** | **High** | ***Previous:***  **leuprolide, bicalutamide**  ***Current (with rising PSA):***  **leuprolide, enzalutamide** |
|  | **2** | **73** | **14.4** | **3.5** | **Bone** | **High** | ***Previous:***  **leuprolide, bicalutamide**  ***Current (with rising PSA):***  **leuprolide, enzalutamide** |
| **3** | **1** | **72** | **158** | **0.7** | **Bone, Lungs, Lymph Nodes, Brain** | **High** | ***Previous:***  **leuprolide, docetaxel, abiraterone, focal radiation**  ***Current (with rising PSA):***  **leuprolide, radium-223** |
| **4** | **1** | **71** | **657.0** | **3.1** | **Lungs, Liver, Lymph Nodes, Bone** | **High** | ***Previous:***  **radiation, leuprolide, bicalutamide, enzalutamide, abiraterone, radium-223, focal radiation**  ***Current (with rising PSA):***  **leuprolide, docetaxel** |
| **5** | **1** | **75** | **33.4** | **2.4** | **Bone** | **High** | ***Previous:***  **leuprolide, bicalutamide, focal radiation, enzalutamide, abiraterone, radium-223**  ***Current (with rising PSA):***  **leuprolide, docetaxel** |
| **6** | **1** | **51** | **185.0** | **3.6** | **Bone** | **High** | ***Previous:***  **leuprolide, docetaxel**  ***Current (with rising PSA):***  **leuprolide, abiraterone** |
| **7** | **1** | **76** | **92.8** | **8.9** | **Lymph Nodes** | **High** | ***Previous:***  **prostatectomy, leuprolide, bicalutamide, focal radiation, imatinib*, nilutamide, abiraterone, enzalutamide, docetaxel, CDU-907*, everolimus, carboplatin**  ***Current (with rising PSA):***  **leuprolide** |
| **8** | **1** | **72** | **3.09** | **1.5** | **Lymph Nodes** | **Low** | ***Previous:***  **prostatectomy, focal radiation, leuprolide, bicalutamide**  ***Current (with rising PSA):***  **leuprolide, abiraterone** |
| **9** | **1** | **83** | **68.3** | **2.4** | **Bone** | **High** | ***Previous:***  **bicalutamide, leuprolide, sipuleucel-T, abiraterone, enzalutamide, focal radiation, CPI-444***  ***Current (with rising PSA):***  **leuprolide, radium-223 + atezolizumab*** |
|  | **2** | **83** | **85.2** | **2.4** | **Bone** | **High** | ***Previous:***  **bicalutamide, leuprolide, sipuleucel-T, abiraterone, enzalutamide, focal radiation, CPI-444***  ***Current (with rising PSA):***  **leuprolide, radium-223 + atezolizumab*** |
| **10** | **1** | **83** | **89.3** | **1.1** | **Lymph Nodes** | **High** | ***Previous:***  **prostate radiation, leuprolide, bicalutamide**  ***Current (with rising PSA):***  **leuprolide, abiraterone** |
| **11** | **1** | **52** | **251.0** | **0.9** | **Bone, Lymph Nodes** | **High** | ***Previous:***  **prostate radiation, leuprolide, bicalutamide, enzalutamide, docetaxel, carboplatin, degarelix**  ***Current (with rising PSA):***  **degarelix, abiraterone** |
| **12** | **1** | **69** | **3.7** | **8.0** | **Bone** | **High** | ***Previous:***  **leuprolide, bicalutamide, abiraterone**  ***Current (with rising PSA):***  **leuprolide, enzalutamide** |
| **13** | **1** | **68** | **402.65** | **1.2** | **Bone** | **High** | ***Previous:***  **prostatectomy, high dose bicalutamide*, focal radiation, leuprolide, enzalutamide, docetaxel**  ***Current (with rising PSA):***  **leuprolide** |
| **14** | **1** | **84** | **52.3** | **0.7** | **Bone** | **High** | ***Previous:***  **prostatectomy, radiation, leuprolide, bicalutamide, abiraterone, docetaxel**  ***Current (with rising PSA):***  **leuprolide, carboplatin** |
| **15** | **1** | **85** | **44.6** | **2.1** | **Bone** | **High** | ***Previous:***  **prostate radiation, bicalutamide, leuprolide, nilutamide, enzalutamide, focal radiation, abiraterone, sipuleucel-T**  ***Current (with rising PSA):***  **leuprolide, docetaxel** |
| **16** | **1** | **76** | **49.0** | **0.7** | **Bone** | **High** | ***Previous:***  **prostatectomy, focal radiation, bicalutamide, leuprolide**  ***Current (with rising PSA):***  **leuprolide, enzalutamide** |

**Supplementary Table 1.** Clinical information on 16 male patients with mCRPC and resistance to ARIs who donated the 19 analyzed blood samples, including age, PSA value at time of blood draw, PSA doubling time prior to blood draw, sites of metastatic disease, disease volume and treatment status. *Agent received on a clinical trial. mCRPC: metastatic castration-resistant prostate cancer; PSA: prostate-specific antigen.

| **Patient ID** | **Sample #** | **AR-V7 Detectable in CTCs** | **AR-V7 Detectable in cfRNA** | **ARI with PSA Progression Before or At Time of Blood Draw** | **Time Between End of Previous ARI Treatment and Blood Draw**  **(in months)** | **Subsequent ARI** | **Start of Subsequent ARI**  **(in months post blood draw)** | **Duration of Subsequent ARI**  **(in months)** | **PSA at Start of Subsequent ARI**  **(in ng/mL)** | **Best PSA Response to Subsequent ARI**  **(in ng/mL)** |
| --- | --- | --- | --- | --- | --- | --- | --- | --- | --- | --- |
| **2** | **1** | **-** | **-** | **enzalutamide** | **0**  **(current treatment)** | **abiraterone** | **32** | **6** | **22.8** | **22** |
|  | **2** | **-** | **-** |  |  |  | **29** |  |  |  |
| **6** | **1** | **+** | **-** | **abiraterone** | **0**  **(current treatment)** | **enzalutamide** | **9** | **21** | **54** | **0.8** |
| **8** | **1** | **-** | **-** | **abiraterone** | **0**  **(current treatment)** | **enzalutamide** | **6** | **7** | **9.9** | **7.04** |
| **10** | **1** | **-** | **-** | **abiraterone** | **0**  **(current treatment)** | **enzalutamide** | **37** | **6** | **123.8** | **14.6** |
| **12** | **1** | **-** | **-** | **enzalutamide** | **0**  **(current treatment)** | **darolutamide** | **41** | **6** | **139.1** | **Progression** |
| **13** | **1** | **-** | **-** | **enzalutamide** | **15** | **abiraterone** | **2** | **4** | **1189** | **802** |
| **16** | **1** | **-** | **-** | **enzalutamide** | **1** | **abiraterone** | **0** | **2** | **49.0** | **Progression** |

**Supplementary Table 2.** AR-V7 detectability in blood, timing and duration of previous ARI treatment with rising PSA, subsequent ARI treatment (if received), and best PSA response to subsequent ARI. Only patients who received another ARI at some point subsequent to the blood draw are included in this table.

| **Patient ID** | **Sample #** | **PSMA Detectable in CTCs** | **PSMA Detectable in cfRNA** | **Timing of PSMA-PET Scan**  **(in months post blood draw)** | **Result of PSMA-PET Scan** |
| --- | --- | --- | --- | --- | --- |
| **2** | **1** | **+** | **-** | **49** | **Positive** |
|  | **2** | **-** | **-** | **47** |  |
| **6** | **1** | **-** | **+** | **42** | **Positive** |
| **14** | **1** | **-** | **-** | **6** | **Positive** |

**Supplementary Table 3.** PSMA detectability in blood and PSMA-PET scan results in patients who received a PSMA-PET scan after blood draw.

| **Sample** | | **Cell Counts** | | **Ct Value of Target Gene** | | | |
| --- | --- | --- | --- | --- | --- | --- | --- |
|  |  | **22Rv1** | **WBCs** | **AR** | **AR-V7** | **CD45** | **GAPDH** |
| Pure AR-V7+ Cancer Cells | | 5,000 | 0 | **24.48** | **23.53** | 34.23 | n/a |
|  |  | 50 | 0 | **26.46** | **29.43** | 29.72 | n/a |
| Mixture of 22Rv1 Prostate Cancer Cells & WBCs | | 12 | 0 | **30.02** | **28.77** | n.d. | 22.34 |
|  |  | 60 | 1,000 | **28.13** | **27.47** | 29.38 | 20.79 |
|  |  | 68 | 3,000 | **31.65** | **29.23** | 29.76 | 21.41 |
|  |  | 6 | 1,000 | **32.55** | **31.45** | 29.67 | 23.14 |
|  |  | 9 | 3,000 | **31.01** | **31.57** | 32.7 | 22.92 |
|  |  | 0 | 1,000 | **n.d.** | **n.d.** | 29.83 | 24.59 |
| Cancer Cells Spiked into Blood from HD and Processed with VTX-1 | Cycle 1 | 16 | 649 | **28.7** | **30.1** | 30.6 | 22.2 |
|  | Cycle 2 | 10 | 245 | **30.2** | **31.0** | 29.9 | 22.1 |
|  | Cycle 3 | 8 | 181 | **31.0** | **30.3** | 31.7 | 22.8 |
|  | cfRNA from HD | n/a | | **33.2** | **n.d.** | n.d. | 22.6 |
| Positive Control | | n/a | | **24.91** | **25.35** | 25.00 | 26.12 |
| No Template Control | | n/a | | **n.d.** | **n.d.** | n.d. | 33.26 |

**Supplementary Table 4.** Mean Ct values of targeted genes from duplicate measurements of pure cancer cells, cell mixtures, cells spiked into blood and processed with VTX-1, and controls. Ct: cycle threshold; WBC: white blood cell; AR: androgen receptor; AR-V7: AR splice variant 7; GAPDH: glyceraldehyde 3-phosphate dehydrogenase; HD: healthy donor; cfRNA: cell-free RNA; n/a: not applicable, since not determined; n.d.: not detected.

| **Patient ID** | **Sample #** | **CTC Subcategories by Immunostaining** | | | | **Total # CTCs** | **# WBCs** | **Blood Volume**  **(in mL)** |
| --- | --- | --- | --- | --- | --- | --- | --- | --- |
|  |  | **DAPI+ CK-**  **VIM- CD45-** | **DAPI+ CK-**  **VIM+ CD45-** | **DAPI+ CK+**  **VIM- CD45-** | **DAPI+ CK+ VIM+ CD45-** |  |  |  |
| **1** | **1** | **92** | **n.d.** | **3** | **n.d.** | **95** | **1786** | **8** |
|  | **2** | **95** | **n.d.** | **1** | **n.d.** | **96** | **2144** | **8** |
| **2** | **1** | **12** | **n.d.** | **0** | **n.d.** | **12** | **2063** | **8** |
|  | **2** | **29** | **0** | **6** | **1** | **36** | **3270** | **8** |
| **3** | **1** | **50** | **n.d.** | **25** | **n.d.** | **75** | **1708** | **8** |
| **4** | **1** | **11** | **n.d.** | **1** | **n.d.** | **12** | **2506** | **8** |
| **5** | **1** | **26** | **0** | **13** | **2** | **41** | **2337** | **8** |
| **6** | **1** | **36** | **0** | **1** | **0** | **37** | **3698** | **8** |
| **7** | **1** | **5** | **0** | **2** | **0** | **7** | **2517** | **8** |
| **8** | **1** | **18** | **0** | **4** | **0** | **22** | **1734** | **8** |
| **9** | **1** | **21** | **0** | **2** | **1** | **24** | **1702** | **8** |
|  | **2** | **19** | **0** | **1** | **0** | **20** | **1110** | **8** |
| **10** | **1** | **45** | **2** | **3** | **0** | **50** | **1569** | **8** |
| **11** | **1** | **4** | **10** | **4** | **2** | **20** | **2384** | **8** |
| **12** | **1** | **50** | **0** | **2** | **0** | **52** | **2378** | **8** |
| **13** | **1** | **9** | **0** | **0** | **8** | **17** | **3642** | **8** |
| **14** | **1** | **14** | **0** | **0** | **3** | **17** | **2257** | **8** |
| **15** | **1** | **61** | **0** | **7** | **0** | **68** | **3519** | **8** |
| **16** | **1** | **10** | **0** | **0** | **0** | **10** | **822** | **8** |

**Supplementary Table 5.** Enumeration of CTCs and WBCs in patient blood samples processed with VTX-1. For definition of CTCs, see Methods section. CTCs: circulating tumor cells; WBCs: white blood cells; DAPI: 4′,6-diamidino-2-phenylindole; CK: cytokeratins; VIM: vimentin; n.d.: not determined (samples were not stained for vimentin).

| **Patient ID** | **Sample #** | **CTCs** | | | | | | **Exosomal cfRNA** | | | | | |
| --- | --- | --- | --- | --- | --- | --- | --- | --- | --- | --- | --- | --- | --- |
|  |  | **AR** | **AR-V7** | **PSMA** | **PSA** | **CD45** | **GAPDH** | **AR** | **AR-V7** | **PSMA** | **PSA** | **CD45** | **GAPDH** |
| 1 | 1 | 26.92 | 31.16 | 26.51 | 32.06 | 25.00 | 16.75 | 28.95 | n.d. | 33.62 | 30.49 | 29.09 | 19.32 |
|  | 2 | 26.16 | 32.25 | 27.09 | 30.36 | 24.66 | 18.16 | 25.80 | 33.36 | 29.16 | 31.47 | 28.98 | 19.31 |
| 2 | 1 | 29.02 | n.d. | 31.50 | n.d. | 25.16 | 20.59 | 32.62 | n.d. | n.d. | n.d. | 33.11 | 22.50 |
|  | 2 | 33.43 | n.d. | n.d. | n.d. | 31.73 | 22.89 | n.d. | n.d. | n.d. | n.d. | n.d. | 24.55 |
| 3 | 1 | 30.21 | n.d. | n.d. | n.d. | 24.65 | 19.81 | 30.20 | n.d. | n.d. | 33.24 | 28.49 | 21.50 |
| 4 | 1 | 28.81 | n.d. | 27.13 | 28.01 | 24.42 | 18.87 | 30.41 | n.d. | 31.20 | 31.38 | 29.97 | 20.89 |
| 5 | 1 | 33.53 | n.d. | n.d. | n.d. | 29.85 | 21.89 | n.d. | n.d. | n.d. | n.d. | 34.69 | 24.17 |
| 6 | 1 | 29.30 | 34.86 | n.d. | n.d. | 30.93 | 20.38 | n.d. | n.d. | 32.97 | n.d. | 34.46 | 23.15 |
| 7 | 1 | 34.21 | n.d. | n.d. | n.d. | 26.45 | 19.81 | n.d. | n.d. | 32.80 | n.d. | n.d. | 22.81 |
| 8 | 1 | n.d. | n.d. | n.d. | n.d. | 29.53 | 21.57 | 32.46 | n.d. | n.d. | n.d. | 31.28 | 21.90 |
| 9 | 1 | 32.99 | n.d. | 33.80 | n.d. | 33.71 | 21.01 | 34.98 | n.d. | n.d. | n.d. | 33.43 | 23.63 |
|  | 2 | 32.16 | n.d. | n.d. | n.d. | 27.83 | 21.68 | 31.00 | n.d. | 31.07 | n.d. | 28.73 | 21.29 |
| 10 | 1 | 31.25 | n.d. | n.d. | n.d. | 30.84 | 22.87 | 34.24 | n.d. | n.d. | n.d. | n.d. | 24.00 |
| 11 | 1 | 31.59 | n.d. | n.d. | n.d. | 29.79 | 20.72 | 33.86 | n.d. | n.d. | 34.17 | n.d. | 24.57 |
| 12 | 1 | 31.97 | n.d. | n.d. | n.d. | 30.27 | 21.07 | 33.79 | n.d. | n.d. | n.d. | 34.87 | 23.59 |
| 13 | 1 | n.d. | n.d. | n.d. | n.d. | 31.62 | 23.73 | n.d. | n.d. | n.d. | n.d. | n.d. | 24.83 |
| 14 | 1 | n.d. | n.d. | n.d. | n.d. | 24.87 | 20.27 | 30.47 | n.d. | n.d. | n.d. | 32.78 | 22.35 |
| 15 | 1 | 31.61 | n.d. | n.d. | n.d. | 24.83 | 18.17 | n.d. | n.d. | n.d. | n.d. | 30.46 | 21.02 |
| 16 | 1 | 33.62 | n.d. | n.d. | n.d. | 30.76 | 22.06 | n.d. | n.d. | n.d. | 31.40 | 34.89 | 24.62 |

**Supplementary Table 6.** Mean Ct values of amplified genes from duplicate measurements in patient blood CTCs and exosomal cfRNA. Ct: cycle threshold; CTCs: circulating tumor cells; cfRNA: cell-free RNA; AR: androgen receptor; AR-V7: AR splice variant 7; PSMA: prostate-specific membrane antigen; PSA: prostate-specific antigen; GAPDH: glyceraldehyde 3-phosphate dehydrogenase n.d.: not detectable

**
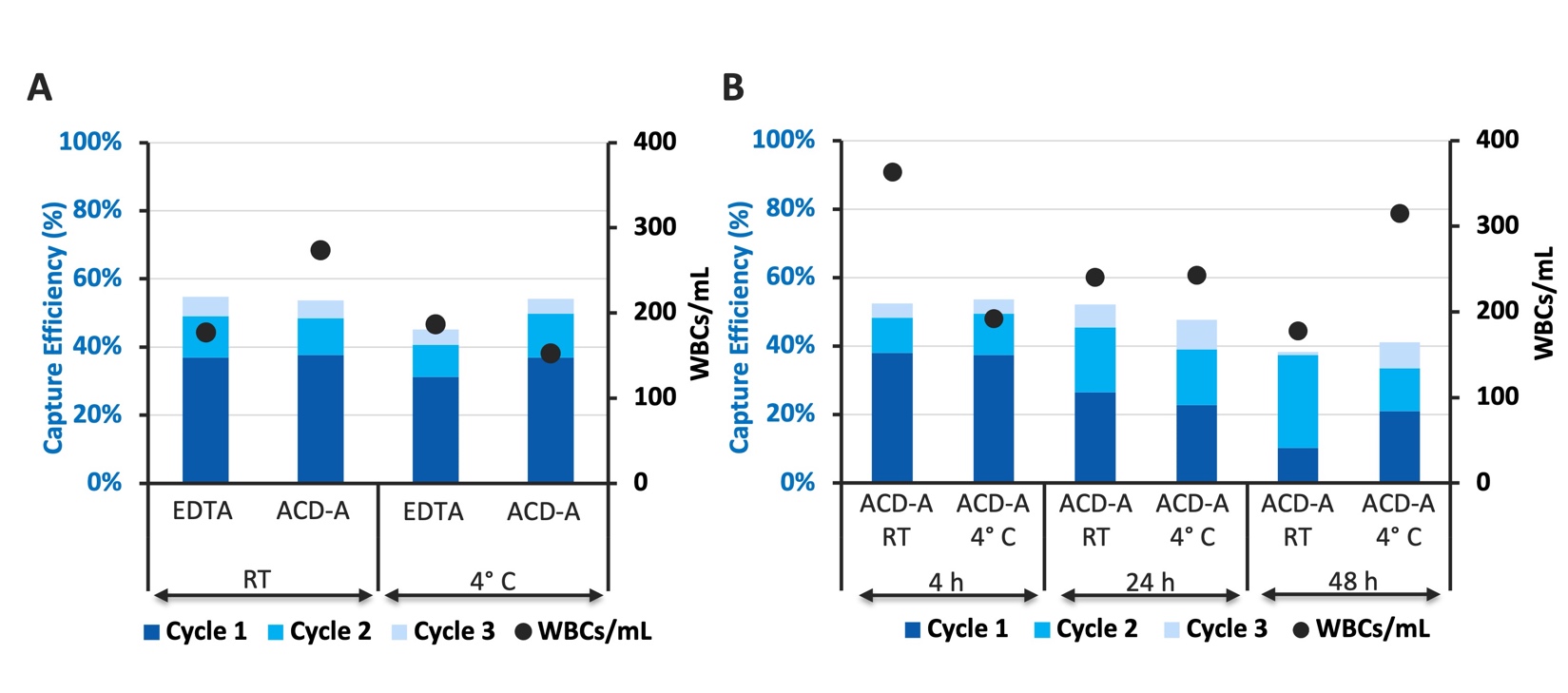
**

**Supplementary Figure 1: Impact of blood tubes and storage temperatures on VTX-1 cell capture efficiency and purity.** For benchmarking against previous VTX-1 performance data, ~500 human MCF7 breast cancer cells were trypsinized, counted, and spiked into 4 mL of whole blood from healthy donors immediately before processing. The suspension was then diluted 10-fold and processed for 3 cycles with the VTX-1 in high-recovery mode. Enriched cells were collected in a well, stained, and enumerated to evaluate capture efficiency and purity.

(A) Blood collected from the same donor in EDTA and ACD-A tubes, stored at RT versus 4˚ C, was processed on the same day and the performance compared side-by-side. ACD-A showed comparable CTC capture efficiency to EDTA at both 4˚ C and RT storage. ACD-A performed similarly at both temperatures (53.7% for RT versus 54.2% for 4˚ C) and was selected for further experiments per QIAGEN AdnaDetect recommendations.

(B) Blood drawn into ACD-A tubes was stored either at RT or 4˚ C for different storage times (4 h versus 24 h versus 48 h post spiking). Capture efficiency remained approximately the same within 24 h, both at RT and 4˚ C (52.5% vs. 53.7% after 4 h, 52.2% vs. 47.8% after 24 h), but decreased by 10% after 48 h (38.3% vs 41.1%).


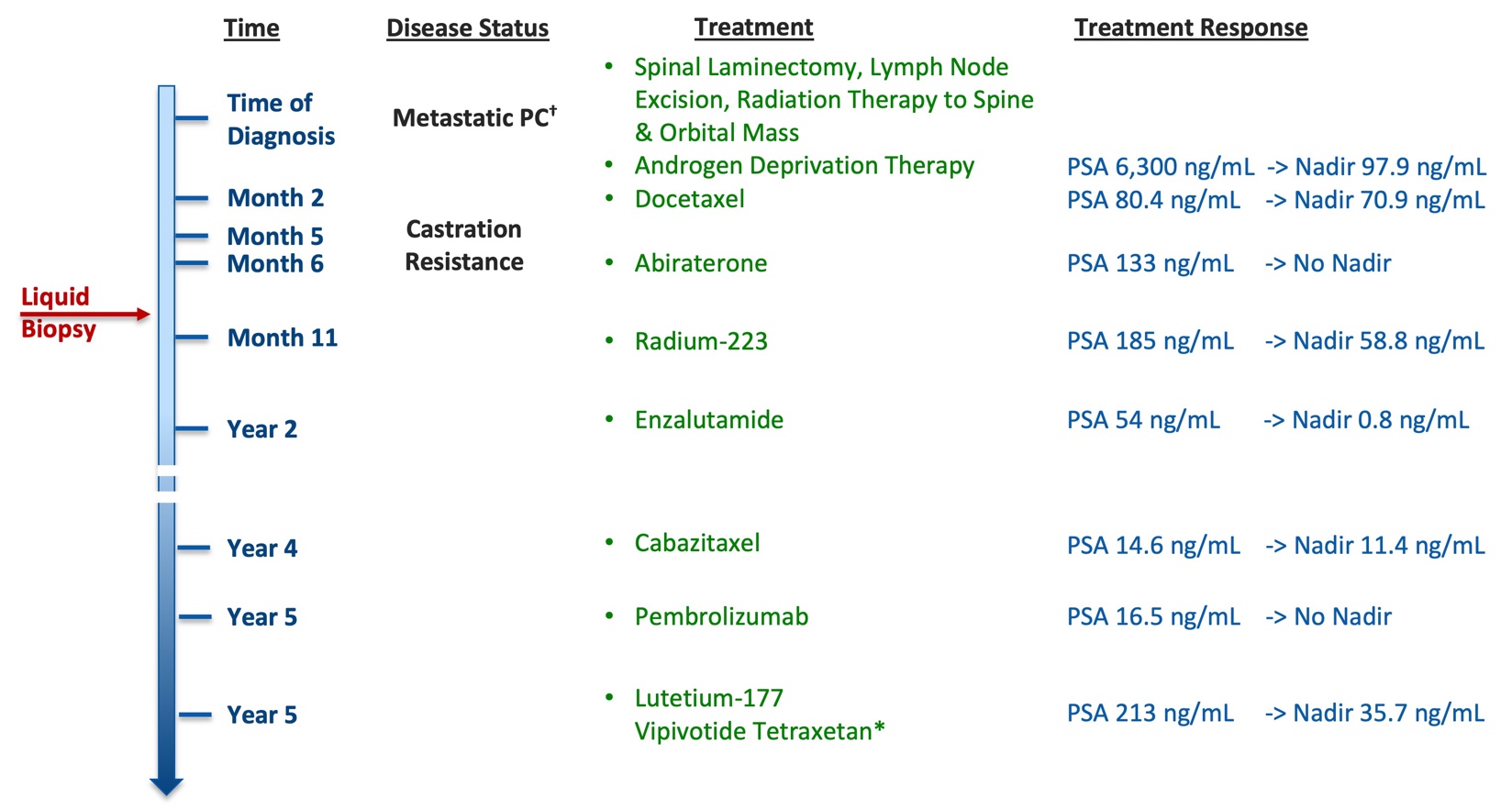


**Supplementary Figure 2. Patient case study.** Clinical course of patient #6 and timing of blood sampling with respect to his treatments and PSA responses. PC: prostate cancer; PSA: prostate-specific antigen. †*de novo* metastatic high tumor burden castration-sensitive prostate cancer. *Patient completed a total of 4 cycles before self-discontinuing due to side effects.
